# Molecular docking analysis of timepidium with Acetylcholine and lumacaftor with GABA(A) activator

**DOI:** 10.6026/97320630015832

**Published:** 2019-12-16

**Authors:** Warda Ali, Nisar A Shar

**Affiliations:** 1 Department of Biomedical Engineering, NED University of Engineering and Technology Karachi, Pakistan

**Keywords:** Epilepsy, repurposing, docking, scoring, electrostatic interaction, seizures

## Abstract

Epilepsy is a chronic disorder characterized by disturbed tissue related molecular activity within the brain irrespective of age. The cause is very difficult to understand towards a suitable treatment. However, its symptoms like seizures are treated and suppressed by known medications. Moreover, the condition is linked with neuro-transmitters such as GABA (gamma amino butyric acid) and acetylcholine. Therefore, it is of interest to design and develop inhibitors for these targets. Hence, we describe the molecular binding features of timepidium with acetylcholine and lumacaftor with GABA(A) activator using molecular docking based geometric optimization and screening analysis for further consideration.

## Background

Epilepsy is a neurological condition in which a person experiences recurrent and unprovoked seizures within a day [1].There are different causes of Epilepsy. The most common cause of Epilepsy is disturbance in the activity of Acetylcholine and GABA. In normal state, GABA act as inhibitory neurotransmitter and Acetylcholine is excitatory neurotransmitter. They regulate cortical function of brain including attention, learning, memory, sleep-wake alternation, and are implicated in neurodegenerative diseases [2].It implies that both GABA and Acetylcholine are important for normal cognitive functions [3].These neurotransmitters also play significant role in sleep deprivation [4], direct coding in retina [5] and in age related hearing loss [6].In epileptic patients, the activity of GABA is suppressed whereas the activity of Acetylcholine is greatly increased [7].However, in case of schizophrenia, the levels of GABA are increased. It has been found that the compounds derived from plants are used to inhibit the level of GABA [8].Different drugs are known to activate the selected neurotransmitters like Emamectin and Ivermectin. Emamectin directly activates acetylcholine and GABA receptors whereas Ivermectin activates GABA (A) receptor only [9].Herbal compounds are also known to activate GABA receptor such as Rosmarinic Acid and Kaempferol [10].

The treatment of epilepsy after occurrence of first seizure is a controversial issue because the underlying mechanisms of brain damage and processes that lead to the development of epileptic conditions are still unknown. However, many successful anti-epileptic drugs AED's have been developed to control seizures; which is one of the most common conditions of epilepsy. These drugs mainly include brivaracetam [11], topiramate [12], phensuximide [13] and fingolimod [14]. AED's stop seizures in approximately 70% of people by controlling chemical activity in brain but they do not cure epilepsy. A study was conducted to check the drug resistance in epileptic patients. If drugs are not effective then seizure activity may be treated either by ketogenic diet [15] or by surgery [16]. It investigated the use of complementary and alternative medicine (CAM) among epileptic patients. It also analyzed the impact of CAM on AED's. The results showed that there is less association between AED's and use of CAM [17].

## Methodology

### Protein target and ligand structures:

The first step was extraction of three-dimensional structures of drugs and proteins. The 3D structures of GABA activators and the program database files (PDB) of Acetylcholine inhibitors were downloaded from Drug Bank Database for docking. The protein ID's for the chosen proteins were obtained from Uniprot. These IDs were then used as an input to download PDB structure of Acetylcholine and GABA receptors from protein data bank. Different receptor chains of Acetylcholine and GABA were analyzed. However, on the basis of their functional properties, six receptor chains of Acetylcholine and four chains of GABA were selected. 45 drugs were randomly selected for Acetylcholine while 47 were selected for GABA. Acetylcholine and GABA recognized some of these drugs while others were unrecognized.

### Electrostatic interactions calculation:

The next step was calculation of electrostatic interactions. SCORE, is used to calculate the electrostatic interactions between the protein as receptor/target and drug. These electrostatic interactions were calculated between randomly selected recognized and unrecognized drugs and target proteins i.e., extracted protein chains of Acetylcholine and GABA. For Acetylcholine, one drug is interacted with three chains (out of six chains) whereas in case of GABA three drugs are interacted with three chains (out of four chains). These interactions are shown in Table 1.

### Molecular docking analysis:

Selecting the highly negative interactions between receptor and ligand using publically available Docking Server and Hex software performed the molecular docking. Chimera was used to visualize results of docking between protein chains and drugs. When drug binds to its target, it releases binding free energy. The binding free energies of the ligand and proteins were computed by using the compute energy tool of the Swiss PDB viewer. The docking server was then used to validate the post docking results. Motifs and domains of the receptor protein were then obtained using SCANPROSITE and ProDom. Examining motifs and domains of the considered protein then did a comparison of the active sites. The residues that lie between the sequence of the motifs and chains were considered as best docked results.

## Results and Discussion:

The results of scoring show positive and negative electrostatic interactions between drugs and their targets. The negative electrostatic interaction indicates more possibility of drug binding with protein. Table 2 indicates that 21 drugs, which are not recognized for Acetylcholine, interacted with all chains of Acetylcholine. It was found that out of these 21 drugs, the best-interacted drug is Timepidium, which gives highly negative electrostatic interaction. However, in case of GABA, 27 drugs that were not recognized by GABA interacted with the chains of GABA. Only 3 drugs show interactions with 3 chains of GABA. The drug Lumacaftor was selected from them as it gave highly negative interaction with the chains of GABA. Hydrogen bonds are weak interactions and important for stabilizing the protein structure in open conformational environment with ligand [18]. Among all highly negative electrostatic interaction of drugs and proteins, hydrogen bonds were observed only in two selected drugs i.e., Timepidium and Lumacaftor.

**Figure 1:**
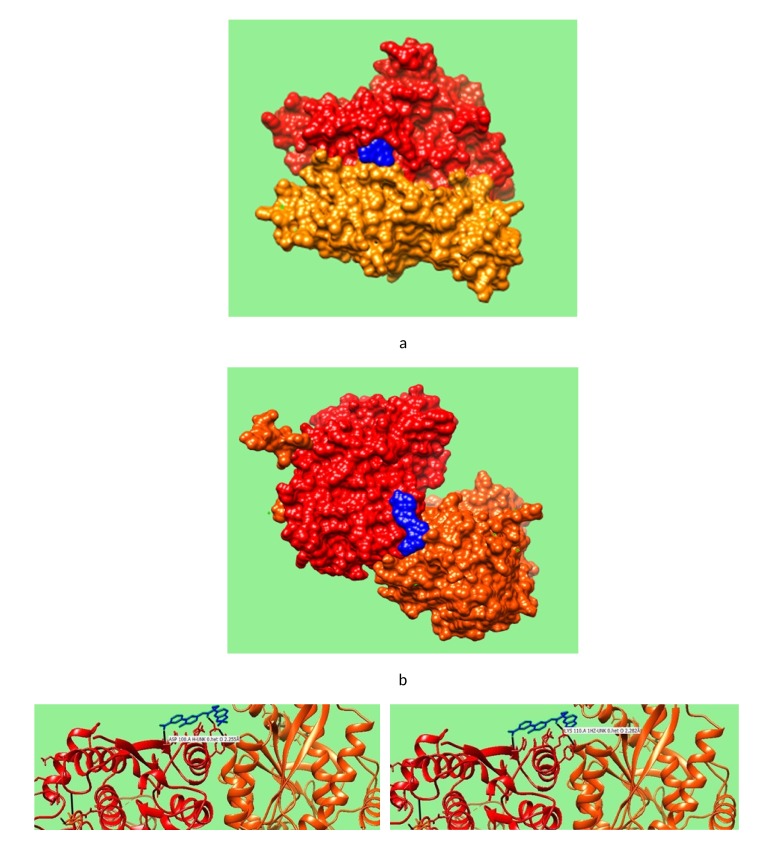
The results of docking between extracted proteins and their targets. Figure 1(a) depicts the Solid model result of Docked Acetylcholine Receptor Alpha unit 2 with Timepidium drug along with an interactive ribbon docked model showing hydrogen bonds. Figure 1(b) depicts the Solid model result of docked Gamma Amino butyric Acid (GABA) Receptor (4MQE) with drug Lumacaftor from CHIMERA. Figure 1(c) further shows two hydrogen bonds as interactive ribbon docked models. It is evident that the bonds in 1(a) are formed between Glutamine residue of Chain-A and Oxygen atoms of the drug with a distance of 2.264 Angstrom whereas in 1(c) the bonds with Aspartate and Lysine residues of Chain A with a distance of 2.255 Angstrom and 2.282 Angstrom respectively. All figures follow a common legend, blue represents the drug chains, red represents Chain-A of Acetylcholine/Chain B of GABA receptor and the orange red represent Chain E of Acetylcholine Receptor/Chain-A of GABA receptor.

The results of docking are shown in Figure 1. In a number of docking results, Timepidium has produced the best result of docking with Acetylcholine receptor 5FJV instead of 6CN. Figure 1a shows that there is only one hydrogen bond between Glutamine residue of Chain A of 5FJV and Oxygen atom of the drug. The distance of this hydrogen bond was found to be 2.264 Angstrom. It indicates best interaction among all docked results. The drug Lumacaftor is found to be well docked with the binding site GABA receptor chain (4MQE). It was found that this interaction was strong forming two hydrogen bonds. The distance of one hydrogen bond between Aspartate residue of Chain A and Oxygen atom of the drug was 2.255 Angstrom whereas other hydrogen bond between Lysine of Chain A and Oxygen atom of Drug had a distance of 2.282 Angstrom.

The results of docking indicate maximum hydrogen and polar bonds between acetylcholine chain with Timepidium and GABA with Lumacaftor. The hydrogen and polar bonds formed between the receptor-ligand complexes of Acetycholine receptor and Timepidium are shown in Table 3. The different types of bond linkages indicate the best-docked results of GABA receptor and Lumacaftor. Table 3 also gives Hydrogen and polar bonds between the oxygen atom of Lumacaftor and the GABA chains. Thus, the presence of hydrogen and polar bonds validate the acquired results. Table 4 is showing binding free energies of drug and their targets. The negative value of the binding energy of the protein-ligand complex is preferred for binding of ligand with its desired protein. It was observed that 4MQE gave highly negative binding free energy with Lysine A whereas 5FJV showed negative interaction with Glutamine B.

Table 5 indicates the predicted binding sites of the chains of the studied proteins. Comparing predicted binding sites of their Motifs and Domains then checked the presence of active sites in Acetylcholine and GABA receptor binding sites. The results indicate that all the binding sites of Acetylcholine receptor chain (5FJV) and GABA receptor chain (4MQE) are present in their corresponding Domains. However, in other chains of GABA and Acetylcholine, the binding sites were not matched in motifs and domains.

## Conclusion

Epilepsy is known to be linked with neuro-transmitters such as GABA (gamma amino butyric acid) and acetylcholine. Therefore, it is of interest to design and develop inhibitors for these targets. However, it is known that lumacaftor has been used to treat Cystic fibrosis (CF) [19] and timepidium bromide to treat abdominal diseases [20]. Hence, it is of importance to evaluate and describe the molecular binding features of timepidium with acetylcholine and lumacaftor with the GABA(A) activator using molecular docking based geometric optimization and screening analysis for further consideration in this context.

## Figures and Tables

**Table 1:** : Total interactions of recognized and not recognized drugs with Acetylcholine and GABA receptor chain to show the electrostatic interactions among drugs and receptor chains

	Total Number of Drugs	Number of Positive Interactions	Number of Negative Interactions	Negative Interaction Value Distribution					
				Drug interacting with all six chains	Drug Interacting with five Chains	Drug Interacting with four Chains	Drug Interacting with three Chains	Drug Interacting with two Chains	Drug Interacting with one Chains
Drugs Recognized By acetyl choline	24	91	53	3	2	2	1	4	6
Drugs Not Recognized By acetyl choline	21	107	19	0	0	0	1	6	4
Drugs Recognized	20	52	28	-	-	0	2	7	8
By GABA									
Drugs Not Recognized	27	66	42	-	-	3	3	9	3
By GABA									

**Table 2:** Drugs docked with Acetylcholine and GABA receptor chains and their binding energy values derived after scoring

	Selected and Docked drugs for Acetyl Choline Chains		
		Drugs	Electrostatic Interactions
	CHRNA 2. 5FJV	Timepidium	-113.6015 Kcal/mol
Acetylcholine Chains	CHRNA4.6CN	Timepidium	-254.0600 Kcal/mol
	CHRNA.2LLY	Timepidium	-198.3092 Kcal/mol
	CHRNA.2LLY	Emetonium iodide	-132.9165 Kcal/mol
	CHRNB5.KXI	Imipramine oxide	-216.1342 Kcal/mol
	CHRNA4.6CN	Bifemelane	-17.4069 Kcal/mol
	CHRNB2.KSR	3,4-Dihydroxybenzoic Acid	-158.9715 Kcal/mol
	CHRNA.2LLY	Tretamine	-114.1014 Kcal/mol
	4MQE	Dactinomycin	-62.2064 Kcal/mol
GABA Chain	4MQE	Lumacaftor	-122.4524 Kcal/mol
	4MQF	Dalfopristin	-89.5999 Kcal/mol

**Table 3:** Hydrogen and polar bonds formed between the Acetylcholine receptor and Timepidium drug and GABA receptor and Lumacaftor drug

Interaction	Hydrogen Bond	Polar Bond
Acetylcholine receptor and Timepidium Drug	N1 - GLU328	O3 - SER84
		N1 - LYS332
		O1 - LYS332
GABA receptor and Lumacaftor Drug	O9 - SER 84	O7 - ASP81
	O8 - ASN 323	N4 - SER84
		N1 - ASN323
		H1 - ASN323
		N3 - ASN 323
		N6 - LYS332

**Table 4:** Binding Free Energy value computation for protein-ligand interactions

	Residue	bonds	Angles	Torsion	Improper	Non bonded	Electrostatic	Total KJ/mol
4MQE	Asp A 100	2.267	4.954	4.939	0.522	-37.12	-0.03	-32.469
4MQE	Lys A 110	5.933	5.013	7.861	0.008	-48.26	-5.97	-35.405
5FJV	Glu A 128	3.592	4.308	11.824	0.059	-6.42	0.57	13.932
5FJV	Glu B 128	4.401	5.236	8.342	1.663	-31.14	1.72	-9.774

**Table 5:** Comparison of target sites of acetylcholine and GABA receptor proteins through motifs and domains by Scan Prosite and ProDom web server

Examine Target Sites Of Protein			
Motifs	Domains	2D Drug Protein Docking Plot	Active Sites From Literature Review (Uniprot 2019)
		(Predicted active sites)	
6CNK			
155 to 169	265 to 524	122,178,179,219,222,000	Not available
5FJV			
161 to 175	62 to 240, 243 to 620	84,136,141,146,147,100,000,000,000	Not available
5KXI			
133 to 144	36 to 214, 217 to 344	136,138,147,148,212	84, 136, 144
135 to 149			
2LLY			
No Hits	7 to 144	Absent	84, 136, 144
2KSR			
No Hits	No Hit	Absent	84, 136, 144
4MQE			
No Hits	37 to 230, 260 to 423	81,84,319,322,323,324,328,332,354	247, 270, 287, 367, 395, 466
4MQF			
No Hits	37 to 230, 260 to 423	81,84,324,328,332	247, 270, 287, 367, 395, 466
